# Prenatal chromium exposure and risk of preterm birth: a cohort study in Hubei, China

**DOI:** 10.1038/s41598-017-03106-z

**Published:** 2017-06-08

**Authors:** Xinyun Pan, Jie Hu, Wei Xia, Bin Zhang, Wenyu Liu, Chuncao Zhang, Jie Yang, Chen Hu, Aifen Zhou, Zhong Chen, Jiangxia Cao, Yiming Zhang, Youjie Wang, Zheng Huang, Bin Lv, Ranran Song, Jianduan Zhang, Shunqing Xu, Yuanyuan Li

**Affiliations:** 1 Key Laboratory of Environment and Health (HUST), Ministry of Education & Ministry of Environmental Protection, and State Key Laboratory of Environmental Health (Incubation), School of Public Health, Tongji Medical College, Huazhong University of Science and Technology, Wuhan, Hubei People’s Republic of China; 2 Women and Children Medical and Healthcare Center of Wuhan, Wuhan, Hubei China

## Abstract

Few studies have investigated the association of environmental chromium exposure and preterm birth in general population. This study was designed to investigate whether maternal chromium exposure during pregnancy is associated with reduced gestational age or risk of preterm birth using the data from Healthy Baby Cohort study conducted in Hubei, China between 2012 and 2014 (n = 7290). Chromium concentrations in maternal urine samples collected at delivery were measured with inductively coupled plasma mass spectrometry. Tertiles of chromium concentrations was negatively associated with gestational age in multivariable linear regression analyses [β (95% CI): low = reference; middle = −0.67 days (−1.14, −0.20); high = −2.30 days (−2.93, −1.67); *p* trend <0.01]. Logistic regression analyses also indicated that higher maternal chromium [adjusted odds ratio (OR) (95% CI): 1.55(0.99, 2.42) for the medium tertile; 1.89(1.13, 3.18) for the highest tertile; *p* trend <0.01] was associated with increased risk of preterm birth. The associations appeared to be more pronounced in male infants (adjusted OR (95% CI): 2.54 (1.29, 4.95) for the medium tertile; 2.92 (1.37, 6.19) for the highest tertile; *p* trend <0.01). Our findings suggest maternal exposure to higher chromium levels during pregnancy may potentially increase the risk of delivering preterm infants, particularly for male infants.

## Introduction

Preterm birth, defined as delivery before 37 weeks of completed gestation, is a leading cause of infant mortality and significant precursor to future morbidity. Every year, about 15 million babies are born prematurely—more than one in 10 of all babies born around the world^1^. Although medical conditions and nutritional status have greatly improved, the rate of preterm birth has not obviously declined. In the U.S., the preterm delivery rate is 12–13%; in Europe and other developed countries, reported rates are generally 5–9%^2^; in Africa, the rate is 11.9%^3^. A variety of risk factors have been linked to preterm birth including medical conditions of the mothers or fetus, genetic influences, behavioral and socioeconomic factors and iatrogenic prematurity^2^, and the maternal exposure to environmental contaminations has been considered as an important contributing factor.

Chromium is a transition metal that is naturally dispersed in the environment, and is primarily in two forms, trivalent (chromium 3+) and hexavalent (chromium 6+). The trivalent chromium is human required, which is directly involved in carbohydrate, fat, and protein metabolism^4–6^, while hexavalent chromium is a toxic metal and has been classified as a human carcinogen. At least 74 million people living in more than 7000 communities in the U.S. drink tap water polluted with chromium^7^. China is a major producer of chromium and the total atmospheric emissions of chromium have increased at annual growth rate of 8.8%^8^. Environmental contamination with chromium is a major threat to human health and has been increasing due to the wide range of industrial uses of chromium globally. Chromium exposure may lead to serious health problems, including oxidation–reduction, protein denaturation and abnormal enzymatic activity^9, 10^. The epidemiological studies reported frequent health problems in chromium exposed population such as, cancer, dermatitis, asthma, chronic bronchitis, hypertension, chromosomal aberrations, back pains, metabolic syndrome, and hemoglobin changes^11–13^.

Chromium can transfer through the placenta to developing fetuses^14^. In animal studies, high doses of chromium exposure during pregnancy impair embryonic development, implantation^15^, and leads to reduced fetal weight, retarded fetal development, skeletal defects, malformations, dead and fetus resorptions^16–18^. The current knowledge concerning the effect of chromium exposure on human health has largely been based on data from occupationally exposed people. Although some studies have demonstrated a significantly increased risk of congenital malformations, low birth weight and DNA damage with infants born to residents living near chromium contaminated areas^19–21^, there is little information on the developmental effects of early life chromium exposure in general population.

In the present study, we conducted a prospective birth cohort study to evaluate the association between maternal chromium exposure during pregnancy and the risk of preterm birth among 7290 pregnant women in Hubei province, China.

## Methods

### Study design and study population

The study populations were selected from the Healthy Baby Cohort (HBC) study in China, a longitudinal birth cohort study of environmental exposures and children’s health. Pregnant women admitted to the hospital for labor have been asked to participate in the study. The eligibility criteria for participants are as follows: (1) residence in Wuhan City at the time of the recruitment period with an expectation to reside continually in this area for the foreseeable future; (2) with a single gestation and live birth; and (3) ability to comprehend the Chinese language and complete the questionnaire. Participants were invited to provide blood and urine samples and to attend face-to-face interviews. Between September 2012 and October 2014, 11,311 women were recruited at Women and Children Medical and Healthcare Center of Wuhan city in central of China. Exclusion criteria included without urine samples available for analysis (n = 3952), and those who gave birth to an infant with a birth defect (n = 57). For the women who had two times delivery in our cohort (n = 3), we chose the first delivery record. In this study, we also excluded 9 women who were smoking (n = 7) and drinking (n = 2) during pregnancy and finally chose 7290 women as our study population.

### Ethics statement

The research protocol was approved by the ethics committee of the Tongji Medical College, Huazhong University of Science and Technology (No. [2012]07), and Women and Children Medical and Healthcare Center of Wuhan (No. 2012003). All participants provided written informed consent at enrollment, and all methods were performed in accordance with the relevant guidelines and regulations.

### Data collection

The face-to-face interviews were conducted with the participants within three days before or after delivery by specially trained nurses in the hospital. The interviews collected a variety of information, including demographic socioeconomic characteristics (e.g., maternal age, education, occupation, household income, and self-reported weight before pregnancy) and lifestyle factors during pregnancy (e.g., smoking, passive smoking, and alcohol consumption). Information about the history of pregnancy outcomes and diseases of mothers, and information concerning the birth date, gender, gestational age at birth and birth weight of infants were retrieved from medical records. The body mass index of mothers was calculated using the self-reported weight before pregnancy and height, which was measured using a stadiometer. Preterm birth was defined as delivery before 37 completed weeks of gestation. The gestational age at delivery was calculated in completed weeks from the first day of the last menstrual period.

### Urine collection and Chromium measurement

The maternal urine samples were collected during admission to the hospital as part of the preparation for delivery. Each subject provided clean and midstream spot urine in a polypropylene collection cup before delivery. The urine specimens were placed in the refrigerator immediately after collection and promptly separated into 5 mL aliquots. All of the urine samples were stored in polypropylene tubes at −20 °C until further analysis.

Prior to analysis, urine samples were thawed at room temperature, and 0.5 mL of urine was introduced in polypropylene conical centrifuge tubes. Then, 1.2% HNO_3_ was added to the final volume of 2.5 mL for overnight nitrification. The resulting sample was dissolved by ultrasound at 40 °C for 1 h and then analyzed for chromium by inductively coupled plasma mass spectrometry (ICP-MS) (Agilent 7900, Agilent Technologies). The operation conditions of ICP-MS were: RF power 1550 w, plasma gas flow 15.00 L/min, auxiliary gas flow 0.8 L/min, carrier gas flow 0.25 L/min, resolution (peak high 10%) 0.65~0.80 amu, improve quantity of samples 0.4 mL/min, unimodal residence time 0.1 s.

The Standard Reference Material Human Urine (SRM2670a Toxic Elements in Urine, National Institute of Standards and Technology, USA) was used as an external quality control in each batch to assess the instrument performance. The control samples were analyzed for elements after calibration and after every 20th sample, and the concentrations measured were within the certified range recommended by the manufacturer (5%). If concentrations were significantly different from the certified value of SRM2670a, the instrument was recalibrated and the previous batch of samples was reanalyzed. A 1.2% HNO_3_ blank was processed in each batch of samples to control for possible contamination. The limit of detection (LOD) for chromium was 0.01 µg/L. The chromium measurements were repeated three times and the average value was used for all statistical analyses. Chromium was detected in 99.2% of the urinary samples in the study. Values below the LOD (n = 62) were replaced with the LOD divided by the square root because this method is used by the CDC^22^ and produces reasonably nonbiased estimates^23^.

Urine creatinine concentrations were determined by a creatinine kit (Mindray BS-200 CREA Kit, Shenzhen Mindray Bio-medical Electronics CO., LTD). Chromium concentrations in urine (µg/L) were adjusted for creatinine in order to account for variations in urine dilution in spot urine specimens, and results were expressed as µg/g cr.

### Statistical analysis

We examined the frequency distributions of maternal sociodemographic characteristics, lifestyle factors, medical and reproductive histories, and infant gender. We used χ2 tests to compare selected characteristics between preterm and term births. The distribution of creatinine-corrected, urinary chromium concentration was right-skewed by the Kolmogorov-Smirnov normality test. The Wilcoxon test was used to compare chromium concentrations between male and female infants. We used a natural log-transformation (ln-Cr) to diminish the influence of extreme values on the regression coefficients. Maternal urinary chromium was also categorized into low (≤1.09 μg/g cr), middle (1.09–3.76 μg/g cr), and high (>3.76 μg/g cr) tertiles. Multivariable linear regression was used to model the relation between continuous ln-Cr or tertile of maternal urinary chromium and each continuous gestational age (days). Then we ran logistic regression to fit the models using maternal urinary chromium concentrations as categorical variables, based on the tertile distribution of chromium concentrations in term births, and the lowest tertile was assigned as the referent group. In these models, we used preterm birth as outcome variable. Further adjustment was based on a priori selection of known risk factors for preterm birth and on results from a forward step-wise model selection procedure with inclusion in final models if they altered urinary chromium concentration effect estimates by >10%. In this study, the three variables that may represent socioeconomic status (SES) including household income (≥50,000 or <50,000 yuan per year), maternal education (more than high school, high school, less than high school) and employment during pregnancy(yes or no), were weakly correlated (Pearson correlation coefficients were r = 0.26 between education and income, and r = 0.35 between education and employment). The likelihood ratio test was used to assess model fit, and inclusion of all three SES variables (education, income, employment) into the model did not significantly improve the model fit compared to addition of each individual variable into the model separately. We selected education to adjust for SES in this study because its adjustment showed a larger impact on the ORs for the association between chromium and preterm birth than the other two SES variables.

In the final models, we adjusted for potential confounding variables including maternal age (<25, 25–29, ≥ 30 years), education (more than high school, high school, less than high school), pre-pregnancy BMI (maternal pre-pregnancy body mass index)(weight (kg)/height(m)^2^)(<18.5, 18.5–23, ≥ 24), parity(1 or ≥ 2), and pregnancy-induced hypertension (yes or no). Additional adjustment for passive smoking during pregnancy (yes or no), gestational diabetes mellitus (yes or no), and infant sex(male or female) did not result in material changes in the observed associations and thus were not included in the final models. Several metals that may be associated with adverse birth outcome (lead, arsenic, cadmium, vanadium and thallium) were also adjusted for in the models to control potential confounding. We also performed sensitivity analyses that excluded maternal urine samples with creatinine <0.3 g/L or >3 g/L^24^, and pregnancy-induced hypertension during pregnancy.

All analyses were performed using the Statistical Package for the Social Sciences (SPSS), version 18.0 (SPSS Inc., Chicago, IL, USA). All statistical tests were considered to be significant at an alpha level of 0.05 for a two-tailed test.

## Results

Of the 7290 singleton live births, the average gestational age was 39.2 ± 1.2 weeks and 3.9% were preterm with a mean gestational age of 35.5 ± 1.2 weeks (Table 1). The mean age of all the participating mothers was 28.5 ± 3.7 years old. About half of the women were in the range from 25 to 29 years old. 67.2% of the women had more than high school educational attainment. More than half of the women had a normal pre-pregnancy BMI. More than 80% of the women were primiparous. There were 3891 male infants and 3399 female infants. Women who had preterm deliveries were more likely to be either younger than 25 years of age or older than 30 years of age, have lower educational attainment, be unemployed during pregnancy, have lower household income and be parous. Women who were diagnosed pregnancy-induced hypertension were also more likely to have preterm birth. Distributions of pre-pregnancy BMI, infant gender, passive smoking, and gestational diabetes mellitus were similar between preterm and term deliveries. The geometric mean of creatinine-adjusted chromium concentration in maternal urine was 2.26, 2.17, and 5.88 µg/g cr in all samples, term birth and preterm birth, respectively. There were no significant differences between the creatinine-adjusted chromium levels of maternal urine samples from the boys’ and girls’ mothers (*p* > 0.05).

**Table 1:** Distribution of Selected Characteristics of the Study Population (n = 7290).

Characteristics	Total(n = 7290)	Preterm(n = 287)	Full-term(n = 7003)
	n(%)	n(%)	n(%)
Maternal age, year*(mean ± SD)	28.5 ± 3.7	29.1 ± 4.5	28.5 ± 3.7
<25	805(11.0)	41(14.3)	764(10.9)
25–29	3985(54.7)	124(43.2)	3861(55.1)
≥30	2500(34.3)	122(42.5)	2378(34.0)
Education*			
More than high school	4898(67.2)	141(49.1)	4757(67.9)
High school	1389(19.1)	50(17.4)	1339(19.1)
Less than high school	1001(13.7)	96(33.4)	905(12.9)
Missing	2(0.03)	0(0.0)	2(0.1)
Employment during pregnancy*			
No	1393(19.1)	92(32.1)	1301(18.6)
Yes	5897(80.9)	195(67.9)	5702(81.4)
Household income*			
≥50,000 yuan per year	4148(56.9)	121(42.2)	4027(57.5)
<50,000 yuan per year	3021(41.4)	164(57.1)	2857(40.8)
Missing	121(1.7)	2(0.7)	119(1.7)
Pre-pregnancy BMI, kg/m^2^			
(mean ± SD)	20.7 ± 2.8	20.8 ± 2.7	20.7 ± 2.8
<18.5	1527(20.9)	59(20.6)	1468(21.0)
18.5–23.9	4832(66.3)	186(64.8)	4646(66.3)
≥24	910(12.5)	37(12.9)	873(12.5)
Missing	21(0.3)	5(1.7)	16(0.2)
Parity*(mean ± SD)	1.2 ± 0.4	1.4 ± 0.5	1.2 ± 0.4
1	6158(84.5)	191(66.6)	5967(85.2)
≥2	1132(15.5)	96(33.4)	1036(14.8)
Passive smoking during pregnancy			
No	6323(86.7)	249(86.8)	6074(86.7)
Yes	967(13.3)	38(13.2)	929(13.3)
Pregnancy-induced hypertension*			
No	7004(96.1)	250(87.1)	6754(96.4)
Yes	286(3.9)	37(12.9)	249(3.6)
Gestational diabetes mellitus			
No	6574(90.2)	254(88.5)	6320(90.2)
Yes	716(9.8)	33(11.5)	683(9.8)
Infant gender			
Male	3891(53.4)	161(56.1)	3730(53.3)
Female	3399(46.6)	126(43.9)	3273(46.7)
Urinary chromium (μg/L)			
Median(IQR)	1.01(0.61–2.09)	1.89(0.95–4.28)	1.00(0.61–2.02)
Urinary chromium (μg/g cr)			
Median(IQR)	1.86(0.86–5.65)	6.08(2.34–15.32)	1.77(0.84–5.24)

*p < 0.01, SD = Standard deviation, GM = Geometric Mean.

Among all infants, we observed statistically significant decreases in gestational age with increasing maternal urinary chromium (both continuous ln-Cr and tertiles of urinary chromium) (Table 2). One unit increase in continuous ln-Cr approximately equals 3-fold increase in continuous chromium (μg/g cr). Thus, for about 3-fold increase in maternal urinary chromium concentrations, we observed 1.05 days decrease in gestational age in the unadjusted analysis (95%CI: −1.19, −0.91). Adjustment for potential confounders, this association approximately halved decreased [β = −0.68 day, 95%CI: (−0.88, −0.48)] and were similar for male [(β = −0.71 day, 95%CI: (−0.98, −0.44)] and female infants [(β = −0.65 day, 95%CI: (−0.95, −0.36)]. For tertiles of chromium concentrations, we observed reduced gestational age with increasing tertile of maternal urinary chromium[β (95%CI): low = reference; middle = −0.67 day (−1.14, −0.20); high = −2.30 day (−2.93, −1.67); *p* trend <0.01]. The inverse trend across tertiles of maternal urinary chromium and gestational age was only slightly enhanced among male infants[β (95%CI): low = reference; middle = −0.95 day (−1.60, −0.29); high = −2.50 day (−3.37, −1.62); *p* trend <0.01] and attenuated among the female infants [β (95% CI): low = reference; middle = −0.39 day (−1.08, −0.28); high = −2.10 day (−3.01, −1.20); *p* trend <0.01].

**Table 2:** Multivariable linear regression analyses of the association between maternal urinary chromium (μg/g cr) and gestational age (days).

Chromium	All infants			Male infants			Female infants			*p*-value
	n	β (95% CI)^a^	β (95% CI)^b^	n	β (95% CI)^a^	β (95% CI)^b^	n	β (95% CI)^a^	β (95% CI)^b^	
Continuous ln-Cr	7290	−1.05(−1.19, −0.91)	−0.68(−0.88, −0.48)	3891	−1.11(−1.29, −0.92)	−0.71(−0.98, −0.44)	3399	−0.95(−1.16, −0.74)	−0.65(−0.95, −0.36)	0.24^d^
Low (≤1.09)	2428	reference	reference	1292	reference	reference	1136	reference	reference	
Medium (1.09–3.76)	2435	−0.95(−1.43, −0.49)	−0.67(−1.14, −0.20)	1263	−1.20(−1.86, −0.54)	−0.95(−1.60, −0.29)	1172	−0.71(−1.39, −0.03)	−0.39(−1.08, −0.28)	
High (>3.76)	2427	−3.49(−3.97, −3.02)	−2.30(−2.93, −1.67)	1336	−3.78(−4.43, −3.13)	−2.50(−3.37, −1.62)	1091	−3.12(−3.81, −2.43)	−2.10(−3.01, −1.20)	
*p* for trend^c^		<0.01	<0.01		<0.01	<0.01		<0.01	<0.01	0.21^e^

CI = confidence interval.

^a^Unadjusted confounders.

^b^Adjusted for maternal age, education, BMI, parity, pregnancy-induced hypertension and other metals.

^c^ *p*-values for trend across tertiles of maternal urinary chromium.

^d^ *p*-value for ln-Cr*sex interaction.

^e^ *p*-value for Cr tertile*sex interaction.

The unadjusted and adjusted ORs and 95% CIs for preterm birth according to the tertiles of chromium concentrations in maternal urine are shown in Table 3. Compared to the lowest tertile of urinary chromium concentrations, a positive significant trend was found between risk of preterm birth and levels of chromium in the adjusted analysis (OR = 1.55, 95%CI: 0.99, 2.42 for the medium tertile; OR = 1.89, 95%CI: 1.13, 3.18 for the highest tertile; *p* trend <0.01). When 1860 women with creatinine concentrations <0.3 g/L or >3.0 g/L were excluded, we observed the adjusted OR for preterm birth was similarly elevated (adjusted OR = 2.05, 95%CI: 1.13, 3.74 for the medium tertile; OR = 3.04, 95%CI: 1.58, 5.84 for the highest tertile; *p* trend <0.01). We also performed an analysis that excluded the women with pregnancy-induced hypertension, and found that the adjusted OR for preterm birth associated with chromium exposure was essentially unchanged (adjusted OR = 1.46, 95%CI: 0.91, 2.34 for the medium tertile; OR = 1.92, 95%CI: 1.12, 3.29 for the highest tertile; *p* trend = 0.02).

**Table 3:** Logistic regression analyses of the association between maternal urinary chromium (μg/g cr) and preterm birth.

Chromium	Term Births	Preterm Birth			
	n	n	OR^a^(95% CI)	OR^b^(95% CI)	OR^c^(95% CI)
**Total (n = 7290)**					
Tertile1 (≤1.07)	2333	31	Reference	Reference	Reference
Tertile2 (1.07–3.52)	2336	71	2.19(1.43, 3.37)	2.16(1.40, 3.34)	1.55(0.99, 2.42)
Tertile3 (>3.52)	2334	185	5.56(3.75, 8.23)	5.32(3.56, 7.93)	1.89(1.13, 3.18)
*p* for trend			<0.01	<0.01	<0.01
**Excluding urine creatinine** <**0.3 or** >**3 g/L (n = 5430)**					
Tertile1 (≤0.88)	1749	16	Reference	Reference	Reference
Tertile2 (0.88–2.11)	1755	42	2.62(1.46, 4.67)	2.52(1.41, 4.53)	2.05(1.13, 3.74)
Tertile3 (>2.11)	1750	118	7.38(4.31, 12.63)	6.74(3.91, 11.62)	3.04(1.58, 5.84)
*p* for trend			<0.01	<0.01	<0.01
**Excluding pregnancy-induced hypertension (n = 7004)**					
Tertile1 (≤1.07)	2249	28	Reference	Reference	Reference
Tertile2 (1.07–3.53)	2256	58	2.03(1.28, 3.20)	2.00(1.27, 3.17)	1.46(0.91,2.34)
Tertile3 (>3.53)	2249	164	5.66(3.74, 8.57)	5.46(3.59, 8.29)	1.92(1.12, 3.29)
*p* for trend			<0.01	<0.01	0.02

Abbreviations: CI, confidence interval; OR, odds ratio.

^a^Unadjusted odds ratio.

^b^Adjusted for maternal age, educational level, BMI, parity, and pregnancy-induced hypertension.

^c^Adjusted for maternal age, educational level, BMI, parity, pregnancy-induced hypertension, and other metals.

We then conducted stratified analyses by infant gender (Table 4). Among male infants, we observed increased risk with increasing tertiles of maternal urinary chromium (adjusted OR = 2.54, 95% CI: 1.29, 4.95 for the medium tertile; adjusted OR = 2.92, 95% CI: 1.37, 6.19 for the highest tertile; *p* trend <0.01). No clear trend in the risk of preterm birth was observed across tertiles of maternal urinary chromium among female infants (adjusted OR = 0.96, 95% CI: 0.51, 1.80 for the medium tertile; adjusted OR = 1.12, 95% CI: 0.54, 2.35 for the highest tertile; *p* trend = 0.72). A borderline significant interaction was observed with respect to infant sex (*p* for interaction = 0.06).

**Table 4:** Logistic regression analyses of the association between maternal urinary chromium (μg/g cr) and preterm birth, stratified by infant gender.

Chromium	Term Births	Preterm Birth			
	n	n	OR^a^(95% CI)	OR^b^(95% CI)	OR^c^(95% CI)
**Male (n = 3891)**	3730	161			
Tertile1 (≤1.07)	1243	12	Reference	Reference	Reference
Tertile2 (1.07–3.70)	1244	42	3.36(1.76, 6.44)	3.39(1.76, 6.52)	2.54(1.29, 4.95)
Tertile3 (>3.70)	1243	107	8.25(4.47, 15.21)	7.90(4.25, 14.67)	2.92(1.37, 6.19)
*p* for trend			<0.01	<0.01	<0.01
**Female (n = 3399)**	3273	126			
Tertile1 (≤1.07)	1091	19	Reference	Reference	Reference
Tertile2 (1.07–3.40)	1091	30	1.51(0.84, 2.72)	1.45(0.79, 2.65)	0.96(0.51, 1.80)
Tertile3 (>3.40)	1091	77	3.79(2.24, 6.41)	3.54(2.06, 6.08)	1.12(0.54, 2.35)
*p* for trend			<0.01	<0.01	0.72

Abbreviations: CI, confidence interval; OR, odds ratio.

^a^Unadjusted odds ratio.

^b^Adjusted for maternal age, educational level, BMI, parity, and pregnancy-induced hypertension.

^c^Adjusted for maternal age, educational level, BMI, parity, pregnancy-induced hypertension, and other metals.

## Discussion

In the present study, we evaluated the relationship between prenatal exposure to chromium and preterm birth in a birth cohort in Hubei China. We found an increased risk of preterm birth associated with higher maternal urinary chromium levels, and the associations appeared to be more evident in male infants. To the best of our knowledge, the present study is the first cohort study to comprehensively examine the association between maternal chromium exposure during pregnancy and the risk of preterm birth.

According to previous epidemiological studies, infants born to residents living near a hazardous waste landfill including chromium contamination have been associated with increased risk of preterm birth^20^. Paternal exposure to welding fumes containing chromium may increase the risk of preterm delivery^25^. In these studies, exposure pollutants contain a wide range of biologically active substances, not only chromium. A study from Guiyu China compared heavy metals including chromium in placenta from women lived in an electronic waste recycling area with those from a control area where no electronic waste processing occurred, and reported that there was no correlation between chromium in placenta and gestational age^26^. Although epidemiologic study on the association between maternal chromium exposure and the risk of preterm delivery is limited, it was confirmed in animal study that chromium increased preterm labor and decreased full-term labor^27^. Our findings provide evidence of a positive association between maternal chromium exposure and risk of preterm birth in general population with environmental level of chromium exposure. In addition, the adjustment for a range of characteristics, including other metals, which have been suggested to be potentially associated with adverse birth outcome in previous studies^28–32^, only slightly attenuated but did not eliminate the significant associations between maternal urinary chromium levels and gestational age, as well as the risk of preterm birth.

Chromium is a naturally occurring heavy metal that can exist in air, water, soil, and food. In the environment, chromium mainly exists in two different stable oxidation states: trivalent chromium(III) and hexavalent chromium(VI). Trivalent chromium, found in most foods and nutrient supplements, is an essential trace metal required for normal carbohydrate and lipid metabolism with very low toxicity^5^. But the essentiality of chromium as a nutrient is now in doubt^33^. Hexavalent chromium is a heavy metal and much more toxic than trivalent chromium^34^. The International Agency for Research on Cancer (IARC)has classified chromium(VI) as a human carcinogen through the inhalation route of exposure^11^. Both chromium (VI) and chromium (III) can cause adverse effects on fertility, reproduction and embryonic development^18, 35–37^. In both industrial and environmental situations chromium (III) and chromium (VI) can inter-convert, with reduction of chromium (VI) to chromium (III) generally being favored in most environmental situations^38^. The body has several systems for reducing chromium (VI) to chromium (III), and this chromium (VI) detoxification leads to increased levels of chromium (III)^39^. Chromium levels in body fluids, such as urine and serum are reliable markers of exposure to chromium in oxidation states (VI) and (III) and provide a measure of the internalized dose of chromium^40^. So we evaluate prenatal chromium exposure by urinary total chromium analysis.

The total chromium in biological samples is analyzed in many studies because of the biological reduction of chromium (VI) to chromium (III)^41^. The pregnant women in our study had higher levels of urinary chromium (median 1.01 µg/L and 1.86 μg/g cr) than those measured in pregnant women in Australia (median 0.25 µg/L and 0.44 μg/g cr)^42^. Our study population also had higher levels of urinary chromium than those reported in non-occupationally exposed adults from the UK (median 0.35 µg/L)^43^, Belgium (median 0.13 µg/L and 0.11 μg/g cr)^44^, Italy (median 0.10 µg/L)^45^, Austria (median1.08 μg/g cr)^46^, and the USA (median 0.12 µg/L and 0.11 μg/g cr)^47^. These findings suggest a regional difference in human exposure to metals. There are currently limited data on chromium exposure levels in the general population in China, and little previous study has reported the urinary chromium levels in Chinese pregnant women. The levels of urinary chromium in our study were slightly higher than those reported in a recent study of men from an infertility clinic in Hubei province^48^, possibly due to the high excretion of chromium as a physiological response with advancing pregnancy^49^. Chromium is a common pollutant introduced into natural waters mainly due to the discharge of wastewater from relative chromium industries. The emissions of chromium by the discharge of wastewater from chromium industries are highly concentrated in the provinces of the eastern, central and southern regions in China, such as Guangdong, Zhejiang, Jiangsu, Hubei and Hunan^8^. The issue may explain that the populations in our study and in the Zeng *et al*. study^48^ have relatively higher levels of urinary chromium than those reported in developed countries.

Although there is currently limited understanding of the potential mechanism between maternal exposure to chromium and an increased risk of preterm infants, one possible mechanism is oxidative stress caused by chromium^50^. Oxidative stress is described as an imbalance in the production of reactive oxygen species (ROS) and the ability of antioxidant (AOX) defenses to scavenge them^51^. Chromium triggers oxidative stress in the cell through increasing lipid oxidation^52^. Increased oxidative stress may play an important role in preterm birth^53^. In animal studies, chromium VI induced developmental toxicity of placenta through downregulating cell survival proteins and increasing cell death by spatiotemporal modulation of apoptotic signaling^54^. The imbalance between ROS and AOXs induced by chromium VI may be one of the mechanisms which potentially affect placental growth and fetal development and thus increased risk of preterm birth^55^. In the stratified analysis, we found that the associations may vary by infant gender in our study, as the significant association between maternal urinary chromium levels and risk of delivering preterm birth was stronger in male infants than in female. One possible explanation would be that chromium has the effect of environmental estrogen and may interfere with the production of estrogen^56^. And the androgen precursors involved in the production of estrogen may be increased in males^57^ and may facilitate preterm labour^58^. It is plausible that the risk of preterm birth associated with chromium exposure could differ by infant sex.

A strength of our study is the large sample size (n = 7290). In addition, interviews conducted with all participants allowed us to adjust for other potential risk factors for preterm birth, such as educational level, household income and passive smoking. Birth outcomes and maternal complications during pregnancy were obtained from medical records, which minimized potential disease misclassification.

There are some limitations to this study. First, maternal urinary chromium levels were only measured at one spot time before delivery and may not be perfect surrogates for prenatal chromium levels. However, the half-life of chromium in urine in occupationally exposed people has been reported to be 129 months^59^, and urinary chromium as a non-invasive biomarker, is considered to be a relatively reliable indicator to reflect chronic exposure. Second, we did not measure the placenta chromium levels, which may not directly reflect accumulation of chromium in the fetus. Third, we did not measure each form of chromium. Chromium (VI) is much more toxic than chromium (III). In the present study, whether the correlation between chromium exposure and preterm birth could be attributable to relatively high percent chromium (VI) dose still needs future studies to validate.

In conclusion, our study found a significantly positive association between maternal urinary chromium levels and the risk of preterm birth. These findings suggest that exposure to chromium in pregnant women may be an important risk factor in the etiology of preterm birth. Appropriate public health measures need to be implemented to reduce preterm birth related to developmental exposure to environmental pollutants, including chromium.
